# “Needle” Abscess in the Horse

**Published:** 1899-06

**Authors:** 


"Needle" Abscess in the Horse. The patient, a bay gelding, was sent to the hospital on account of a diffuse inflammatory swelling on the inferior abdominal surface. The swelling suppurated and broke in three places. While accidentally drawing the hand over the swelling a sharp point was felt which, on removal with the forceps, proved to be a needle about three inches long with a piece of heavy string in the eye. It was evidently a needle such as millers use in sewing up bags. The horse had evidently swallowed the needle in his feed, and from the intestines it migrated until it found its way to the abdominal surface, where it caused subcutaneous suppuration and pierced the skin. Cicatrization soon followed.

